# Integrating Systems and Critical Thinking, Clinical Reasoning, Decision‐Making, and Judgement in Dietetic Education: Development of a Conceptual Pilot Model

**DOI:** 10.1111/jhn.70343

**Published:** 2026-09-22

**Authors:** Sonja Schönberg, Elisa Bertozzi, Jessica Stalder, Anita Bucher, Joyce Haddad, Adrian Rufener, Sandra Jent

**Affiliations:** ^1^ Department of Health Professions Bern University of Applied Sciences Bern Switzerland

**Keywords:** clinical decision making, clinical judgement, clinical reasoning, cognitive strategies, critical thinking, dietetic education, Nutrition Care Process (NCP), systems thinking

## Abstract

**Introduction:**

Dietetic practice is increasingly characterised by complexity arising from chronic disease, food and nutrition insecurity, demographic shifts, and environmental sustainability concerns. Addressing this complexity requires the effective and interprofessionally aligned use of central cognitive strategies. However, despite frequent reference to systems thinking, critical thinking, clinical reasoning, clinical decision‐making, and clinical judgement in health professional education, inconsistent use, and limited shared understanding across professions hinder coherent educational approaches – particularly in dietetic education, where these strategies underpin the Nutrition Care Process (NCP). This article presents an initiative at a Swiss University of Applied Sciences to develop a conceptual pilot model bringing together these five cognitive strategies for Bachelor‐level nutrition and dietetics education, aiming to clarify their relationships, support their use in teaching the NCP and contribute to reflection on a shared interprofessional language.

**Methods:**

A qualitative case study design with an iterative–inductive reflexive interpretive approach was used to explore shared and profession‐specific understandings of central cognitive strategies within Bachelor‐level nursing, midwifery, physiotherapy, and nutrition and dietetics education. Data were generated through a targeted narrative literature review, consultations with five expert educators across the four professions, and a feedback session with 16 members of the Nutrition and Dietetics Bachelor's educational team. These inputs informed the development of a conceptual pilot model.

**Results:**

The analysis revealed variation in how the five cognitive strategies are defined, emphasised, and applied across dietetics and other health professions. In response, the *Five Cognitive Strategies in Dietetics Education Pilot Model* offers an initial shared vocabulary and positions the strategies in relation to the NCP.

**Conclusions:**

This article introduces a conceptual pilot model addressing fragmented understandings of key cognitive strategies in Bachelor‐level dietetic education. By linking five commonly cited strategies to the NCP, it offers a preliminary framework to support reflection, dialogue, and engagement with complexity in practice. While not intended as a comprehensive or definitive framework, making these strategies explicit may foster a more analytically grounded understanding of professional decision‐making. Further piloting and refinement are needed to assess the model's relevance and educational value.

## Key points

- Rising complexity in dietetics: Contemporary practice requires dietitians to navigate uncertainty and competing demands, making advanced cognitive strategies essential for sound decision‐making.
- Inconsistent use of key concepts: Core strategies – such as systems thinking, critical thinking, clinical reasoning, clinical decision‐making, and clinical judgement – are inconsistently defined and applied within dietetic education and across health professions.
- A pilot model to integrate strategies: The article introduces a conceptual pilot model that brings together five cognitive strategies and links them to the Nutrition Care Process (NCP) to support teaching and curriculum development.
- Supporting shared understanding in education and practice: Explicitly integrating these cognitive strategies may enhance reflection, strengthen professional reasoning, and foster a more coherent interprofessional language, although further validation of the model is required.

## 1 Introduction

Dietetic practice is becoming increasingly complex due to the rising prevalence of chronic diseases, demographic shifts, and environmental sustainability concerns [1]. These developments require dietitians to make justified decisions under uncertainty, balance competing priorities, and respond to adaptive challenges – situations without clear solutions or with multiple possible courses of action [1, 2]. Meeting these demands requires not only nutritional expertise, but also the ability to reason systematically, reflect on professional decisions, and articulate reasoning within interprofessional teams.

To prepare students for these demands, dietetic education must foster such capabilities. A key approach is the use of higher‐order thinking strategies‐referred to here as *cognitive strategies* – including systems thinking, critical thinking, clinical reasoning, clinical decision‐making, and clinical judgement. These strategies are widely cited across health professions as essential for evidence‐informed, structured, collaborative, and reflective practice. However, they are neither coherently conceptualised across professions nor consistently defined within disciplines [3]. Identical terms may denote different cognitive processes in dietetics and other health professions, while similar processes may be labelled differently, hindering interprofessional dialogue and coherent curriculum design.

These conceptual ambiguities challenge interprofessional collaboration and influence how complexity is addressed in dietetics [3, 4]. In dietetic practice, systematic reasoning – central to navigating complex situations – is strongly structured through the Nutrition Care Process (NCP). While the NCP provides an important framework that explicitly incorporates critical thinking and clarifies its relevance within dietetics, other cognitive strategies are less articulated [5, 6]. For instance, clinical reasoning and clinical decision‐making – well‐established in disciplines such as nursing, midwifery, and physiotherapy – remain comparatively under pronounced [3, 7]. Clinical judgement is implicitly addressed but not conceptually foregrounded; for example, Vo et al. [8, 9] describe it as extending beyond critical thinking and decision‐making, emphasising its multidimensional and context‐dependent nature. In addition, recent scholarship highlights systems thinking as essential for addressing adaptive challenges within food and health systems, with calls to embed it throughout the NCP, similar to critical thinking [5, 10]. This uneven representation contributes to a fragmented understanding of cognitive strategies, potentially limiting their systematic integration in dietetic education. Addressing these gaps is therefore particularly relevant in interprofessional healthcare education.

In response to increasing complexity and the need for transversal competencies [11], the Faculty of Health Professions at a *(details anonymised)* University of Applied Sciences undertook an interprofessional revision of its bachelor's curricula. This resulted in a shared educational framework comprising 12 focus areas, linked to interprofessional and profession‐specific graduate competencies. One overarching focus area is critical thinking, encompassing competencies such as decision‐making, judgement, systematic thinking, and interprofessional discourse.

However, the heterogeneity of cognitive strategies described within and across professions raised critical questions regarding how these central forms of cognition can be conceptualised, aligned, and made explicit in health professions education. During this process, the nutrition and dietetics education team identified a particular challenge in working with the NCP. Specifically, they were concerned that inconsistent conceptualisations of cognitive strategies may limit students' development of metacognitive awareness in process‐oriented work and hinder the establishment of a shared professional language for explaining and justifying professional decisions.

Therefore, the present study sought to establish a more systematic and transparent conceptual foundation to support the development of cognitive strategies that prepare students for professional practice. Emerging from an ongoing curriculum development process at this institution, the study reflects a practice‐oriented and iterative engagement with these challenges. It explores how five commonly referenced cognitive strategies – systems thinking, critical thinking, clinical reasoning, clinical decision‐making, and clinical judgement – are understood and addressed across health professions within this University, and how they may be more coherently articulated within Bachelor‐level nutrition and dietetics education.

Informed by a Qualitative Health Research (QHR) orientation [12], the study aimed to generate practice‐relevant conceptual insights while informing ongoing curriculum development. Guided by the Emphasis–Purposeful sample–Phenomenon of interest–Context (EPPiC) framework [12], the research question was: *How, and informed by which literature, are central cognitive strategies taught by educators in nursing, midwifery, physiotherapy, and nutrition and dietetics, and how can these be aligned and integrated into the NCP to foster a shared professional language within a Bachelor's programme in nutrition and dietetics?*

The article presents a pilot model as a preliminary instrument to support reflection, dialogue, and curriculum development within Bachelor‐level nutrition and dietetics education, by enhancing transparency regarding how cognitive strategies are understood, taught, and articulated.

## 2 Methods

### 2.1 Study Design

**FIGURE 1:**
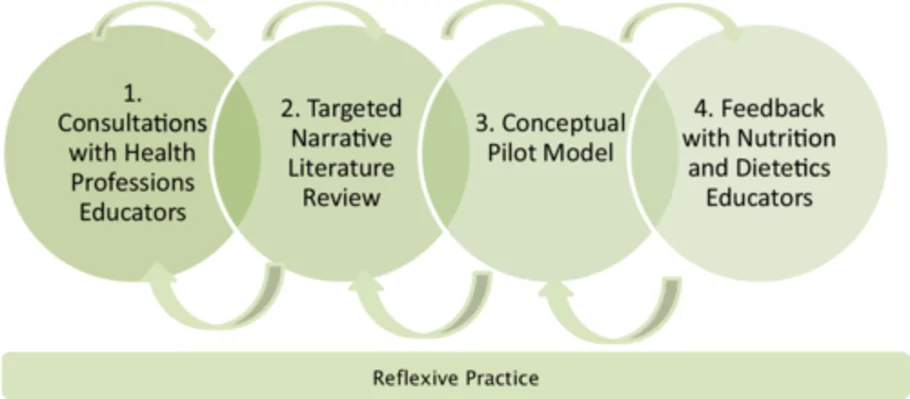
Visualisation of the iterative–inductive research process for the conceptual pilot model.

Drawing on Swift and Tischler [13], the research strategy of an exploratory qualitative case study was derived from the research question. Qualitative case study research is considered particularly valuable in applied nutrition science, as it allows in‐depth exploration of phenomena – here educational practices and programme development – in real‐world settings [12]. The study design was guided by the assumption that explicating implicit and explicit cognitive strategies for navigating complexity requires iterative engagement with multiple perspectives and sources. Accordingly, it integrated consultations with health professions educators, literature engagement, and a feedback session with nutrition and dietetic educators, aiming to develop a conceptual pilot model of the interrelations among cognitive strategies, see Figure 1. These sources were treated as mutually informing rather than sequential, following an iterative–inductive logic [14]. The study drew on key elements of this approach – learning through ongoing enquiry, inductive immersion, sensitising, iterative sense‐making, and reflexivity – without formal coding or a comprehensive analytic framework. Data generation and synthesis proceeded in parallel: Insights emerging from each source informed subsequent discussions and data collection steps, and the evolving focus of interest in an iterative manner.

The study was conducted by seven nutrition and dietetics scholars who contributed to data collection, synthesis, and writing. Two senior S.J. and J.H. and one junior scholar S.S. had formal training and some experience in qualitative research and contributed methodological perspectives as needed. One senior scholar A.R. and three junior scholars E.B., J.S., and A.B. conducted the literature engagement, consultations with health professions educators, and organised the nutrition and dietetics educators' feedback session. The Consolidated Criteria for Reporting Qualitative Research (COREQ) guidelines [15] served as a reference point that informed reporting of the study.

### 2.2 Setting and Participants

The study was conducted between March and August 2025, at a Faculty of Health Professions at a Swiss University of Applied Sciences offering bachelor's, master's, and continuous education programmes in nursing, midwifery, physiotherapy, and nutrition and dietetics, alongside applied research in these fields. At the time of the study, the school had 90 faculty members in nursing, 47 in midwifery, 73 in physiotherapy, and 43 in nutrition and dietetics employed. Bachelor's student enrolment comprised 401 nursing, 226 midwifery, 329 physiotherapy, and 172 nutrition and dietetics students.

The interprofessional bachelor's curriculum revision, initiated in 2022 and planned for implementation in fall 2027, aims to strengthen the health care system through improved education, with critical thinking defined as one key focus area. This prompted discussions within the nutrition and dietetics team about how critical thinking is understood, operationalised, and taught across disciplines, highlighting the need to clarify concepts, align goals, and articulate perspectives, including their integration into the NCP.

Participants involved across the different study components were purposively selected. The consultations included five in‐house educators from Bachelor's programmes in nursing (*n* = 1), midwifery (*n* = 2), physiotherapy (*n* = 1), and interprofessional education (*n* = 1). The educators were chosen based on their recognised expertise in teaching cognitive strategies in their curriculum. No other educators with explicit expertise in this field were known to the authors. Of those contacted, only one midwifery educator referred the invitation to a colleague with more advanced expertise in the field. The feedback session involved 16 members of the nutrition and dietetics Bachelor's educational team with a total of 37 members. The only criterion for inclusion of the nutrition and dietetics team members was that they had a teaching role at the time of the feedback session.

### 2.3 Data Collection

Data collection included targeted consultations with health professions educators, a targeted narrative literature review and a feedback session with nutrition and dietetics educators, all facilitated by E.B., J.S., A.R., and S.S.

#### 2.3.1 Consultations With Health Professions Educators

After the literature review had been initiated, targeted consultations with the health professions educators were conducted between April and May 2025. Consultations involved oral (face‐to‐face or online) and written exchanges. While email correspondence and sharing of profession‐specific curricular documents and literature constituted the primary source of substantive material, oral exchanges supported clarification and exploration of initial assumptions and the refinement of emerging ideas. Brief field notes were taken and subsequently expanded through team reflection.

Initial exchanges focused on how critical thinking is understood, taught, and operationalised within different health professions. In parallel, an ongoing review of relevant literature sensitised the research team to emerging concepts, leading to expanded enquiry into clinical reasoning, clinical decision‐making, and systems thinking. Subsequent research team discussions explored how these concepts are defined, interrelated, and embedded within professional curricula. All materials received from the consultations, together with the related correspondence, were archived in a dedicated electronic folder. Key information was then extracted, synthesised, and organised to enable comparison across health professions and provide a structured overview of the collected data.

#### 2.3.2 Narrative Literature Review

A targeted narrative literature review was conducted between March and May 2025 as part of the study's iterative conceptual development process. A systematic review approach was not considered appropriate given the exploratory and interpretive aims of the study, which focused on conceptual clarification and curriculum development rather than comprehensive evidence synthesis or effect evaluation. The review was undertaken to inform the conceptualisation and definition of five cognitive strategies and to support interpretation of empirical findings.

The review initially focused on critical thinking, clinical reasoning, and clinical decision‐making, and was later expanded to include clinical judgement and systems thinking, following consultation with health professions educators. Sources were identified through targeted searches in PubMed, reference lists, grey literature, and teaching materials provided by participating educators. Search terms combined cognitive strategies (e.g., ‘critical thinking’, ‘clinical reasoning’, and ‘clinical decision making’), dietetic processes (e.g., ‘Nutrition Care Process’, ‘NCP’), and professional contexts (e.g., ‘dietetics’, ‘healthcare professions’, ‘nursing’, ‘physiotherapy’, and ‘midwifery’). Publications in English, German, and Italian were included if they addressed conceptual or empirical aspects of these strategies in dietetics or related health professions. No publication year limits were applied.

Retrieved publications were imported into and organised using Zotero® reference management software (Version 9.0.6, Digital Scholar) to facilitate storage, categorisation, and citation. Retrieved publications were reviewed for their conceptual relevance, educational focus, and contribution to differentiating, or contextualising the cognitive strategies under investigation. Conceptualisation refers to the theoretical framing of the cognitive strategy (e.g., its dimensions, components or attributes); differentiation refers to distinguishing it from related constructs; and contextualisation refers to applying it within a specific professional or educational context.

Given the narrative nature of the review, studies were not formally appraised and no systematic screening procedures were applied. In total, 30 publications informed the final synthesis. A summary of the principal sources underpinning the conceptualisations is provided in Table S1 in the Supplementary Material.

#### 2.3.3 Conceptual Pilot Model and Linking the Five Cognitive Strategies to Dietetic Practice

Building on insights generated through the health professions educators' consultations and the iterative literature review, the next step was to clarify the interconnections between the identified cognitive strategies. Through iterative research team discussions, the development of an initial conceptual pilot model serving as a clarification instrument emerged as a suitable approach to support shared reflection and dialogue. The model synthesised diverse conceptualisations of critical thinking, clinical reasoning, clinical decision‐making, clinical judgement, and systems thinking, alongside a preliminary visualisation of their potential interrelations. Developed as a working artefact, it served as a basis for the feedback session with nutrition and dietetics educators (see Section 2.3.4) and subsequent refinement through reflection on findings from literature and health professions educators' consultation amongst the researchers involved in the present study.

Following development of the initial conceptual pilot model, the five cognitive strategies were illustratively linked to the NCP through iterative research team discussions. The NCP is the standardised framework underpinning dietetic practice, comprising four interrelated steps: nutrition assessment, nutrition diagnosis, nutrition intervention, and nutrition monitoring and evaluation [6]. It was selected as an illustrative framework because of its widespread use in dietetics education and practice in Switzerland and internationally [16].

#### 2.3.4 Feedback Session With Nutrition and Dietetics Educators

An in‐person feedback session on the conceptual pilot model was held with nutrition and dietetics educators from the institutional team as prospective users and a valuable source for input for shaping the model's applicability in teaching.

The feedback session explored the preliminary conceptual pilot model's clarity, relevance, and usefulness for dietetics curricula, as well as current and prospective approaches to teaching the five cognitive strategies. To guide discussion, participants worked in small groups using the following prompts:

- How are the five cognitive strategies understood?
- How are the proposed relationships among these strategies perceived?
- Which of these strategies are already addressed, implicitly or explicitly, in dietetic education?
- Where and how are these strategies currently integrated?
- What ideas exist for future implementation?

Responses were documented on flipcharts and subsequently discussed in plenary.

### 2.4 Data Synthesis and Reflexivity

As this work emerged from an interprofessional curriculum revision, data synthesis did not follow classical case study analytic procedures. Instead, all data sources were integrated through an iterative, interpretive sense‐making process guided by the research question and informed by reflexive qualitative research strategies to enhance trustworthiness [13]. Based on the premise that multiple perspectives can enrich understanding of both a phenomenon and its context [12], the synthesis adopted an interpretive, reflexive stance informed by intuitive enquiry [17], emphasising inductive engagement with the data and the development of conceptual coherence rather than formal thematic categorisation.

Analysis involved iterative–inductive engagement with three interconnected data sources (Figure 1), with synthesis occurring alongside data collection. Field notes from consultations informed team discussions and shaped the literature review, while literature insights were summarised E.B. and J.S., critically reflected upon, and used as sensitising concepts. Feedback from nutrition and dietetics educators, together with all materials, was progressively integrated into a shared working document capturing key ideas, conceptual distinctions, and emerging interpretations.

**FIGURE 2:**
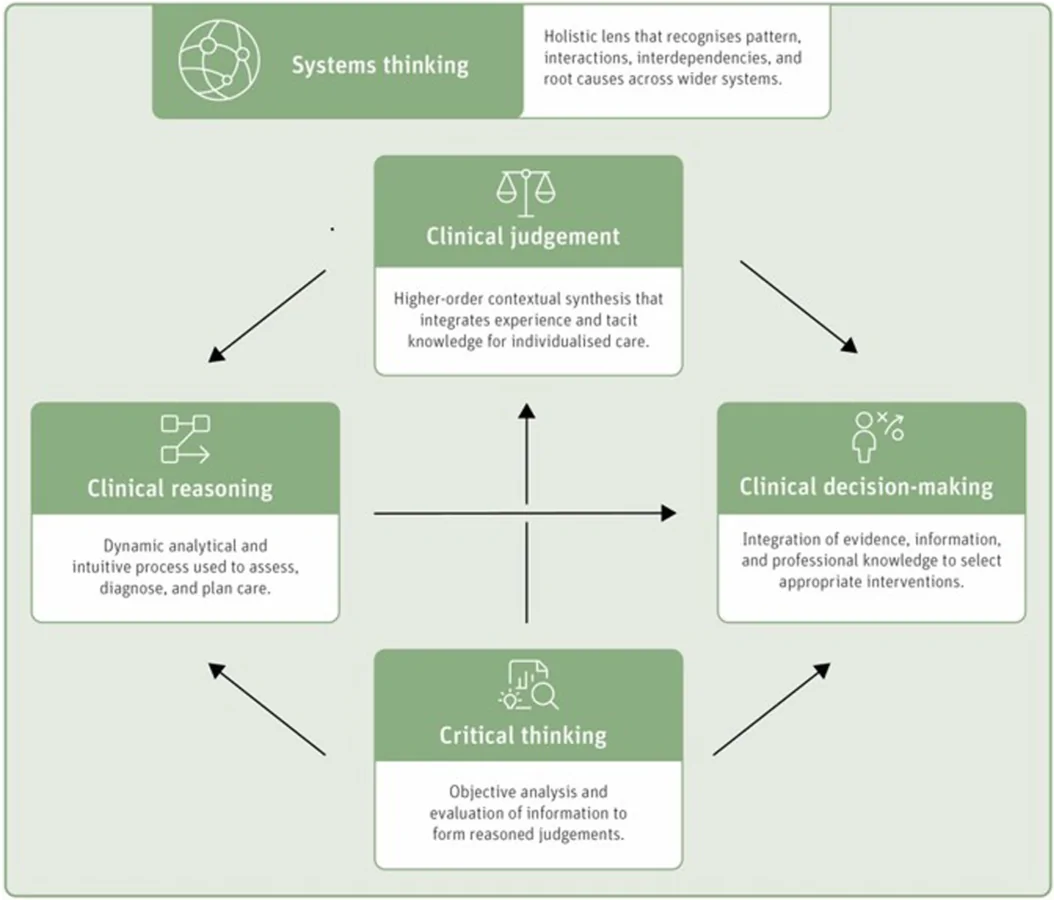
Five cognitive strategies in Dietetics Education Pilot Model illustrating the relationships among five cognitive strategies in dietetic practice. Systems thinking provides the overarching contextual orientation, while critical thinking underpins clinical reasoning, clinical decision‐making, and higher‐order clinical judgement. Arrows indicate the direction the cognitive strategies mainly inform each other.

In the final stage, these iterative insights were systematised into the inductively developed *Five Cognitive Strategies in Dietetics Education Pilot Model* (Figure 2), visualising relationships between cognitive strategies. Given the study's exploratory nature, formal coding was not applied; instead, interpretation was advanced through reflexive team dialogue. Artificial intelligence (ChatGPT, OpenAI) supported refinement of the model's visual representation, while the final layout was developed with support from the institution's professional communications and design team. All conceptual decisions remained with the authors.

Reflexivity was embedded throughout as a continuous orientation, shaping engagement with data, collaborative synthesis, and writing. The research team's expertise in dietetics education and their roles in teaching and curriculum development were treated as analytical resources and made explicit through ongoing reflection, informing both interpretation and model development.

## 3 Results

The results present the synthesised outcome of the iterative–inductive process described above at the level of the integrated conceptual result. Rather than tracing how individual data sources informed each cognitive strategy over time, the analysis reflects the interpretive nature of the enquiry and its exploratory, practice‐oriented aim of achieving conceptual coherence for dietetic education and the NCP within a Swiss University of Applied Sciences.

While alternative conceptualisations are acknowledged, the five strategies – systems thinking, critical thinking, clinical reasoning, clinical decision‐making, and clinical judgement – are presented as reference points for further development, including piloting and evaluation of the model's pedagogical utility.

Findings are reported in two stages. First, synthesised understandings of each cognitive strategy are presented, together with the *Five Cognitive Strategies in Dietetics Education Pilot Model* (Figure 2), which proposes a conceptualisation of the strategies as analytically distinct yet dynamically interrelated within a non‐linear system. Second, illustrative linkages between each cognitive strategy and NCP steps are outlined in Table 1.

**TABLE 1:** Key elements of the five cognitive strategies for each NCP step.

NCP step	Systems thinking	Critical thinking	Clinical reasoning	Clinical decision‐making	Clinical judgement
Assessment	Define roles and tasks in the interprofessional team to ensure effective collaboration. Analyse the client's systemic context and relationships, assessing their impact on health.	Analyse and interpret data (not just collect them). Identify relevant, reliable, and consistent data. Identify missing data. Differentiate symptoms, causes, and contributing factors.	Formulate hypotheses on underlying physiological or behavioural mechanisms. Apply evidence to interpret data. Compare findings with reference values and standards, considering individual context (e.g., medical condition, lifestyle).	Decide whether additional data are required. Prioritise urgent issues.	Assess the data completeness and consistency. Establish which problems are likely nutrition‐related.
Diagnosis	Formulate diagnoses considering systemic factors.	Structure information systematically (e.g., PES structure: Problem – Aetiology – Symptoms). Check validity and plausibility of the hypothesis. Weigh alternative hypotheses.	Link data to diagnosis (causal reasoning). Identify underlying mechanisms.	Determine problem to be prioritised. Define treatment goals and success criteria.	Select most relevant nutrition diagnosis. Evaluate supporting evidence.
Intervention	Plan interventions within the entire systemic context of the individual.	Select evidence‐based, context‐specific interventions. Evaluate alternatives. Anticipate effects under different conditions (prognostic thinking).	Integrate evidence, clinical experience, and patient preferences. Derive individualised recommendations. Anticipate barriers and side effects.	Decide on interventions and sequencing. Define goals, materials, and communication.	Assess feasibility, ethics, and patient‐centeredness of interventions. Evaluate potential for change and patient motivation.
Monitoring and evaluation	Reflect and integrate the systemic context into monitoring and evaluation.	Analyse outcome data. Assess effectiveness, relevance and sustainability. Reflect on the decision‐making process.	Interpret trends and contributing factors. Formulate hypotheses for observed deviations.	Decide to continue, modify, or discontinue intervention. Determine need for further diagnostics.	Adapt the intervention plan based on new insights. Engage in shared decision‐making with the patient.

### 3.1 Emergent Understandings of the Five Cognitive Strategies

#### 3.1.1 Critical Thinking

Critical thinking involves objective analysis, interpretation, and evaluation of information to form a reasoned judgement [5, 18, 19]. Facione [19] conceptualises critical thinking as «purposeful, reflective judgement about what to believe or what to do», comprising six core cognitive skills: interpretation, analysis, inference, evaluation, explanation, and self‐regulation.

Critical thinking underpins all steps of the NCP: ensuring accurate data collection and interpretation during assessment, supporting diagnosis, guiding goal setting and intervention strategies, and enabling outcome evaluation [6]. For dietitians, it involves gathering and analysing information, recognising relationships, synthesising conclusions, and testing hypotheses in care planning, drawing on knowledge, problem‐solving strategies, and reflective evaluation to optimise care [20]. Its development in students is supported by teaching strategies, such as creative assignments, modelling practical situations peer collaboration, student–teacher interaction (with the teacher introducing different perspectives on a problem), and self‐reflection [21].

In this study, critical thinking was the first cognitive strategy examined, reflecting its prominence in the institution's new educational framework and its recognised role in the nutrition and dietetic curriculum (e.g., within NCP).

Educators from all participating health professions educators identified critical thinking as relevant in the consultations, although its conceptualisation and level of implementation varied across disciplines. One, for example, mentioned that all the terms are related, describing different aspects of the same phenomenon. Another educator expressed that they did not have a clear definition of the term critical thinking but saw it potentially as a part of clinical reasoning. In the conceptual pilot model, critical thinking is positioned at the base, representing the foundational cognitive skills that support the other cognitive strategies (Figure 2).

#### 3.1.2 Clinical Reasoning

Clinical reasoning encompasses the cognitive processes used by healthcare professionals during assessment, diagnosis, and treatment to reach sound clinical decisions. In the pilot model, this has been visualised by placing clinical reasoning as a cognitive strategy informing clinical decision‐making (Figure 2). Clinical reasoning integrates analytical thinking with information gathering, perception, and consideration of medical, ethical, and contextual factors. As a dynamic, cyclical process, it supports personalised care and shared decision‐making [22].

The consultations with health professions educators indicated that clinical reasoning was regarded as a key cognitive strategy taught in physiotherapy, midwifery, and nursing, with materials mainly referencing Higgs et al. [23] and Klemme and Siegmann [22]. The teaching materials provided by the educators confirmed the explicit integration of clinical reasoning in the curriculum. Although all cognitive strategies were included in the questions for the group discussions in the nutrition and dietetics educators' feedback session, clinical reasoning was not addressed by any group. This may reflect differences in terminology, emphasis, or professional priorities across the participating disciplines.

Clinical reasoning can follow intuitive or analytical pathways, as described in the Dual Process Framework, which distinguishes fast, experience‐based reasoning from slower, deliberate analysis [24]. Critical thinking forms its foundation [25], as shown in Figure 2. Learning clinical reasoning is challenging, requiring active practice in real patient encounters rather than theoretical knowledge alone. It is best taught within structured frameworks that make reasoning explicit through observation, explanation, and feedback, fostering reflection and improving outcomes [26]. Despite its importance, no consensus definition exists, underscoring the need for research to clarify and measure this competency [27]. Awareness of cognitive biases and reasoning errors is essential to minimise negative impacts on clinical decisions [24].

#### 3.1.3 Clinical Decision‐Making

Clinical decision‐making is the process of selectively integrating information, evaluating evidence, and applying professional knowledge to select appropriate interventions [28]. It represents the concrete decision emerging from the broader clinical reasoning process, grounded in critical thinking [22] and informed by clinical reasoning (Figure 2). This multidimensional phenomenon involves tasks, interactions, reasoning, practitioner factors, and context, all integrated through clinical judgement [9]. Influencing factors include experience, intuition, information management, and clinical context [28]. Experienced practitioners combine analytical and intuitive approaches, including pattern recognition [9]. While evidence‐based medicine has advanced decision‐making, its application to individual cases remains challenging [29]. In our study, clinical decision‐making was mentioned by midwifery and nursing educators, but not by physiotherapy and nutrition and dietetics educators. This observation was further supported by the teaching materials shared by participants. One educator mentioned that other concepts such as participative decision‐making, informed consent, and informed choice were also taught as decision‐making models.

Increasing data volumes and algorithmic tools highlight the need for cognitive strategies to structure and manage information. Clinical decision‐making entails defining criteria, comparing probabilities and benefits, and applying statistical concepts such as conditional probability and Bayes' theorem [30]. When evidence is limited, intuition plays a role but should be supported by critical thinking and biological plausibility to ensure safety [29]. Reflection during and after care is crucial for refining decision‐making and improving outcomes, reinforcing the need for educational strategies that make decision‐making explicit and context‐sensitive.

#### 3.1.4 Clinical Judgement

Clinical judgement is understood as a core component of therapeutic practice, involving complex cognitive processes that integrate diverse sources of information and knowledge. It extends beyond data‐driven reasoning to include narrative and virtue‐based approaches [31]. Clinical judgement also supports treatment evaluation and reflection‐in‐action, incorporating holistic and intuitive understanding of complex situations (Gestalt cognition) [32].

In dietetics, it is conceptualised as a higher‐order, practitioner‐dependent meta‐reasoning process that draws on tacit knowledge and experience to manage complexity, guide interactions, and individualise care [8]. Recent research positions clinical judgement as an overarching cognitive strategy that coordinates technical tasks, interpersonal interactions, practitioner factors, and contextual influences, combining analytical and intuitive reasoning [9]. It is central to patient communication, diagnosis, and therapeutic decision‐making, supporting person‐centred care and professional relationships [8, 32]. To represent its overarching nature, clinical judgement has been positioned above clinical reasoning, clinical decision‐making and critical thinking in the pilot model (Figure 2).

Beyond dietetics, the literature on clinical judgement is predominantly situated within nursing [33], with limited evidence on how it is taught [34]. Across the additional data sources included in this study, clinical judgement was not explicitly mentioned by the participating health professions educators, in the materials provided, or in the nutrition and dietetic educators' feedback session. This may indicate that the term is not explicitly integrated into the institution's curricula.

Nevertheless, the inclusion of clinical judgement in the conceptual pilot model is supported by literature suggesting that, in an era of digitalisation and big data, maintaining a pluralistic approach that integrates data‐driven and experience‐based reasoning remains essential for addressing individual patient complexity [31]. Its contextual sensitivity is strengthened by systems thinking, which broadens the scope of information considered and makes interactions, interdependencies and wider determinants explicit. Clinical judgement operationalises this systems perspective by integrating these factors into patient‐specific situations and clinical decision‐making.

#### 3.1.5 Systems Thinking

Derived from General Systems Theory [35], systems thinking is increasingly recognised in health contexts for its potential to address complex problems by focusing on interactions among system elements and their influence on the system as a whole [36]. Its application spans patient care and organisational structures, aiming to improve quality through understanding dynamic interactions among people, processes, and technologies [37, 38]. Core features include a comprehensive perspective, integrative thinking, pattern recognition, and an appreciation of emergent phenomena [35, 39]. Defined as ‘the ability to recognise and synthesise patterns, interactions, and interdependencies’ [40], for dietitians systems thinking requires critical thinking, problem‐solving, and multi‐perspective analysis [5]. Within the pilot model (Figure 2), systems thinking provides a contextual orientation, framing how complexity is recognised and navigated. Thus, it functions as an overarching orientation informing other cognitive strategies, highlighting how integrative thinking, pattern recognition, and attention to emergent phenomena underpin approaches to complexity in dietetic practice. In the pilot model, all processes are embedded within systems thinking, which integrates and coordinates their relationships and broadens perspective across the wider context, without implying linear sequencing or hierarchy. Systems Thinking enables the identification of root causes of nutrition‐related problems through a determinants‐of‐health lens and makes explicit processes often applied intuitively in expert practice [1]. This systemic perspective informs both clinical reasoning and decision‐making.

Across data sources, systems thinking appeared to be conceptualised in different ways across professions and minimally embedded in nutrition and dietetics. It was not mentioned by health professions educators and was heterogeneously integrated by nutrition and dietetics educators. This aligns with literature indicating that, despite its relevance for complex problem‐solving, systems thinking remains underrepresented in health education and practice [37]. In dietetics, its curricular integration is limited; students struggle to apply it, and educators report low confidence due to limited awareness, resources, and training [5, 41, 42, 43]. By directing attention to interactions among clinical, social, organisational, and structural factors, systems thinking broadens the understanding and delivery of nutrition care and may enhance quality, safety, and outcomes [39]. In line with Bergquist and Tagtow [5], findings support its integration across all steps of the NCP, including social, commercial, and political determinants, highlighting its integrative role in the pilot model and in shaping clinical judgement. Within the pilot model, systems thinking provides the contextual orientation for the other cognitive strategies by drawing attention to interactions among clinical, social, organisational, and structural determinants. Clinical judgement synthesises these system‐level considerations with patient‐specific information, professional experience and tacit knowledge to support contextually appropriate and individualised care.

### 3.2 Linking the Five Cognitive Strategies to the Nutrition Care Process

Table 1 presents example‐based, pedagogically oriented foci for each of the five cognitive strategies. This table is not exhaustive or prescriptive; rather it illustrates how the integrated cognitive strategies may be made visible within a familiar processual framework used in nutrition and dietetic education.

While Figure 2 depicts the conceptual relationships between the five cognitive strategies as analytically distinct constructs, it should not be interpreted as a linear progression. Instead, the model is intended to be situated within the iterative NCP cycle, in which monitoring and evaluation continuously feed back into reassessment. The strategies are therefore understood as dynamically interacting across all NCP steps, with Figure 2 illustrating their relationships rather than the sequence of the process itself.

The assignments presented in Table 1 should be understood as illustrative rather than definitive. They reflect the authors' judgement regarding the primary cognitive emphasis of each activity, recognising that many activities may reasonably be associated with more than one cognitive strategy given the dynamic and overlapping nature of these constructs.

The examples draw on expert educator consultations, literature, and educators' feedback, and are intended to support pedagogical reflection rather than standardisation.

## 4 Discussion

This study explored how five cognitive strategies – systems thinking, critical thinking, clinical reasoning, clinical decision‐making, and clinical judgement – are conceptualised and taught across selected health professions at Bachelor's level within a Swiss University of Applied Sciences, and how they can be more coherently aligned with the NCP in nutrition and dietetics education. Addressing the growing complexity of dietetic practice, findings informed the development of the *Five Cognitive Strategies in Dietetics Education Pilot Model* (Figure 2) as a contextually grounded, reflective tool. The model aims to make cognitive strategies more explicit, pedagogically actionable, and better integrated with the NCP, supporting local curriculum development and contributing to ongoing discussions on educational innovation and systems‐oriented dietetic practice.

### 4.1 Addressing Complexity and Conceptual Coherence in Dietetic Education

Existing dietetics scholarship increasingly positions systems thinking as a core competency for addressing the complexity of contemporary nutrition practice, emphasising pattern recognition, integrative thinking, and the ability of dietitians to recognise and synthesise interrelationships among clinical, social, environmental, organisational, and policy influences on health and nutrition outcomes [1, 5, 40]. Such perspectives support dietitians in identifying root causes of nutrition‐related problems rather than focusing solely on isolated clinical issues. Recent work has advocated for embedding systems thinking throughout the NCP, while highlighting persistent barriers to implementation, including limited educator confidence and awareness, insufficient resources, and difficulties translating systems‐oriented perspectives into teaching and practice [43, 44]. The findings of this study are consistent with these reports, as systems thinking was variably known, conceptualised and appeared only minimally embedded within undergraduate nutrition and dietetics and across health professions education. The overarching role of systems thinking in the *Five Cognitive Strategies in Dietetics Education Pilot Model* (Figure 2) was therefore informed primarily by the literature rather than by current educational practice. This reflects a deliberate design decision, as contemporary dietetics scholarship increasingly positions systems thinking as essential for understanding complexity and for integrating clinical, organisational, social, environmental, and policy influences on nutrition care.

Existing scholarship further suggests that systems thinking relies on critical thinking, problem‐solving, and the integration of multiple perspectives [5]. However, our findings indicate that these relationships remain insufficiently articulated within education, with systems thinking frequently disconnected from other cognitive strategies that support professional action, including critical thinking, clinical reasoning, clinical decision‐making, and clinical judgement. This fragmentation was not only reflected in educator perspectives but also in the literature reviewed. Such a lack of conceptual coherence may complicate undergraduate teaching and hinder the development of a shared language for learning and collaboration. The novelty of the *Five Cognitive Strategies in Dietetics Education Pilot Model* lies less in the identification of new cognitive strategies than in its attempt to provide conceptual coherence by explicitly linking systems thinking, critical thinking, clinical reasoning, clinical decision‐making, and clinical judgement within a single educational framework aligned with the NCP.

The proposed *Five Cognitive Strategies in Dietetics Education Pilot Model* (Figure 2) extends existing work by making relationships between cognitive strategies explicit and by positioning systems thinking as an overarching orientation that informs, connects, and contextualises other cognitive strategies within the NCP. In doing so, it shifts attention from systems thinking as a standalone competency towards its role in supporting more integrated approaches to professional learning and dietetic practice.

Developed within a local curriculum revision, the *Five Cognitive Strategies in Dietetics Education Pilot Model* provides a heuristic framework for discussing cognitive processes that often remain implicit within health professions education. Feedback from nutrition and dietetics educators further highlighted its potential to support teaching, curriculum reflection, and the integration of systems thinking into nutrition and dietetic education.

Taken together, the findings suggest that the five cognitive strategies are taught and conceptualised differently across the health professions included in this study. While critical thinking was recognised across disciplines, clinical reasoning and clinical decision‐making were more explicitly embedded in nursing, midwifery, and physiotherapy education, whereas systems thinking and clinical judgement appeared less consistently articulated within both curricular materials and educator accounts. These differences, together with the diverse literature informing each strategy, reinforced the need for a framework capable of aligning and relating these concepts within nutrition and dietetics education.

However, conceptual coherence alone does not establish professional competence. To understand the educational and professional significance of the five cognitive strategies, they need to be situated within established competency frameworks and linked to the observable activities through which professional thinking becomes visible in practice.

### 4.2 Relating the Five Strategies to Existing Competency Frameworks

Building on the need to move beyond conceptual clarification alone, the *Five Cognitive Strategies in Dietetics Education Pilot Model* must be understood in relation to broader competency frameworks that guide professional education and practice. These include interprofessional frameworks such as CanMEDS and Health Systems Science, as well as profession‐specific competency standards used within nutrition and dietetics education and practice. The model is not intended as an alternative to these frameworks but as a conceptual clarification of cognitive processes that operate across broader domains of professional competence. More specifically, it should be understood as a heuristic educational framework designed to support reflection, teaching, curriculum development, and discussion of cognitive processes within the NCP, rather than as a competency framework, prescriptive curriculum model, or predictive model of professional performance. This positioning is consistent with broader frameworks used across health professions education. Health Systems Science foregrounds the organisational, relational and systems conditions through which care is delivered [45, 46] while CanMEDS organises professional capability through interrelated roles, including Medical Expert, Communicator, Collaborator, Leader, Health Advocate, Scholar, and Professional. As an interprofessional reference framework relevant to the health professions represented in this study, CanMEDS illustrates how the five cognitive strategies may function as cross‐cutting cognitive and metacognitive resources across clinical practice, collaboration, scholarship, leadership, and professionalism rather than as stand‐alone competencies [47].

A similar interpretation is reflected within profession‐specific dietetic competency frameworks. The British Dietetic Association's (BDA) Model and Process for Nutrition and Dietetic Practice embeds clinical reasoning throughout a structured, person‐centred dietetic process and requires the reasoning underpinning interventions to be communicated clearly [48]. The BDA Dietetic Career Framework similarly expresses clinical reasoning, decision‐making and risk management as capabilities that become progressively more complex across levels of practice [49]. Cognitive processes are thus positioned as integral to consultation, documentation, communication, and safe professional performance rather than treated solely as internal mental activities.

Similarly, the National Competency Standards for Dietitians in Australia translate cognition into observable practice. They include the demonstration of clinical reasoning and decision‐making in nutrition care plans and documentation, while the Australian Scope of Practice framework requires practitioners to evaluate and justify whether activities fall within their individual scope of safe practice [50, 51]. This illustrates that professional judgement includes not only choosing an intervention but also recognising personal limitations, seeking appropriate support and deciding when referral or collaboration is required.

In the United States, the Commission on Dietetic Registration's Essential Practice Competencies include a distinct sphere named Critical Thinking and Decision Making. This sphere encompasses professional judgement, strategic thinking, problem‐solving, evidence integration, consultation with others, and awareness of how personal values and biases influence decisions. The framework thereby connects cognitive strategies with observable performance indicators and with wider domains such as ethics, communication, evidence‐informed practice, safety, and quality improvement [52].

None of these frameworks appears to operationalise all five strategies as a coherent and explicitly differentiated set. Rather, individual strategies are variously named, combined within broader competency domains, or implied through performance expectations. Rather than focusing on relationships among cognitive processes, these frameworks primarily describe how cognition becomes visible through competent professional performance. The contribution of the *Five Cognitive Strategies in Dietetics Education Pilot Model* therefore lies not in introducing new competencies, but in making explicit the conceptual connections among cognitive processes that are often embedded within broader competency domains. Across these frameworks, the terminology and organisation of the constructs differ, but a common pattern is apparent: cognition is operationalised through situated professional action; critical thinking supports evidence appraisal and reflection; clinical reasoning supports the interpretation of patient information and generation of possible courses of action; clinical decision‐making concerns the justified selection among alternatives; clinical judgement integrates evidence, experience, ethical responsibility, patient preferences, and scope of practice; and systems thinking connects individual decisions with team, organisational, population, and food‐system contexts. The *Five Cognitive Strategies in Dietetics Education Pilot Model* therefore addresses selected processes underpinning competent action rather than the full range of professional roles, behaviours and capabilities.

Its potential contribution lies in offering a differentiated vocabulary for examining cognitive processes that broader competency frameworks often combine within more encompassing domains. The dietetic frameworks, in turn, indicate how these processes may be translated into developmental expectations, observable activities and assessment criteria. Entrustable Professional Activities (EPAs) may provide one means of achieving this operationalisation by defining authentic units of dietetic practice through which the integration of multiple cognitive strategies, competencies, and professional roles can be observed and assessed [53, 54]. This positioning raises a further question: if the five strategies are understood as cognitive processes rather than competencies in themselves, how do they become visible in professional practice? One way of addressing this question is to distinguish between ways of thinking and ways of practising.

### 4.3 From Ways of Thinking to Ways of Practising

Building on this competency‐based positioning, it becomes necessary to consider how the five cognitive strategies are enacted and demonstrated in practice. While they describe cognitive processes, professional education ultimately requires these processes to be translated into observable forms of performance. Critical thinking and systems thinking can primarily be understood as orientations through which practitioners analyse information, question assumptions, recognise relationships, and frame complex situations. Clinical reasoning, clinical decision‐making, and clinical judgement are more directly situated within clinical activity, but they also remain partly internal processes. They become observable when practitioners collect and interpret information, formulate and revise hypotheses, prioritise nutrition problems, justify an intervention, communicate uncertainty, or adapt a care plan in response to outcomes.

The distinction is therefore analytical rather than categorical. Cognitive processes become professional competencies when they are combined with knowledge, communication, ethical responsibility, and contextually appropriate action. Clinical reasoning curricula consequently emphasise the need to make reasoning explicit through authentic clinical activities, explanation, observation, feedback, and reflection [4]. Competency‐based and entrustment‐oriented approaches similarly assess whether learners can integrate their capabilities in real or simulated professional tasks rather than merely demonstrate knowledge of the relevant concepts [55, 56].

Table 1 provides an initial illustration of this translation by showing how the five strategies may become visible across assessment, diagnosis, intervention, and monitoring and evaluation. In the next phase of curriculum development, these illustrative manifestations are intended to inform the development of EPAs for both university‐based education and practice placements. EPAs may enable the integration of the five cognitive strategies within authentic units of dietetic practice while making expected levels of supervision, responsibility, and performance explicit.

However, observable professional performance is not only individual but also relational and situated within teams and care systems. Clinical reasoning and related cognitive processes are also socially and materially situated rather than exclusively located within an individual practitioner. Clinical reasoning may be distributed across practitioners, patients, professional groups, documentation systems, technologies, and organisational routines. Collaborative clinical reasoning involves sharing information, negotiating interpretations, coordinating different professional perspectives, and developing a course of action that no single participant may have produced independently [57]. The plurality of clinical reasoning traditions across health professions further indicates that professions may attend to different information, use different explanatory models, or describe related cognitive processes using different terminology [58].

For dietetics, this collaborative dimension is particularly relevant because nutrition‐related problems frequently intersect with medical treatment, nursing care, rehabilitation, psychosocial support, food provision, and discharge planning. The purpose of a shared language is therefore not to standardise all professional reasoning, but to enable team members to make their assumptions, interpretations, and uncertainties discussable. In the *Five Cognitive Strategies in Dietetics Education Pilot Model*, collaboration can be understood as a mode of practising through which all five strategies are enacted: critical thinking supports appraisal of competing claims, clinical reasoning supports joint interpretation, decision‐making involves negotiation among alternatives, clinical judgement integrates professional and patient perspectives, and systems thinking draws attention to interdependencies among team members and services [4, 57].

Systems thinking provides the conceptual bridge between individual cognitive processes and strategic, team‐based, and systems‐level practice. Health Systems Science situates clinical care within interacting structures such as care teams, organisational processes, information systems, community contexts, policy arrangements, and population health priorities [45, 46]. From this perspective, a clinically plausible intervention cannot be judged solely in relation to the individual patient; its feasibility, accessibility, continuity, equity, and interaction with other components of the care system also require consideration.

In dietetics, Bergquist and Tagtow [5] propose integrating systems thinking and sustainable food‐systems perspectives throughout dietetics education and across the NCP. Their approach broadens the focus from immediate clinical variables to the social, commercial, political, and environmental determinants shaping nutrition and health. This supports the *Five Cognitive Strategies in Dietetics Education Pilot Model*'s representation of systems thinking as an overarching orientation rather than an isolated step or separate technique. Systems thinking expands the boundaries of critical thinking, clinical reasoning, decision‐making, and judgement by shaping which relationships, consequences, and levels of intervention are considered relevant.

Strategic and integrated problem‐solving can consequently be conceptualised as ways of practising through which the five cognitive strategies are mobilised across levels. At the individual level, a dietitian may adapt an intervention to medical, cultural, and socioeconomic conditions. At the team level, the dietitian may coordinate nutrition goals with other care plans and clarify responsibilities. At the organisational or systems level, repeated difficulties may prompt changes to referral pathways, documentation processes, food provision, or quality‐improvement activities. Such movement between individual cases and wider systems is especially important when practitioners encounter uncertainty and complexity that cannot be managed through isolated clinical decisions [59].

Collaborative, strategic, and integrated problem‐solving should therefore not necessarily be added to the model as further cognitive strategies. They may be more coherently interpreted as observable and situated ways of practising through which the five strategies are combined.

### 4.4 Educational Relevance and Contribution of the Pilot Model

Taken together, the preceding discussion suggests that the educational value of the *Five Cognitive Strategies in Dietetics Education Pilot Model* lies in its ability to bridge conceptual understanding, competency development, and professional practice within the NCP. By explicitly linking systems thinking, critical thinking, clinical reasoning, clinical decision‐making, and clinical judgement, the model provides a coherent framework for understanding how cognitive processes relate to competent dietetic practice.

First, it provides greater conceptual coherence by making relationships among systems thinking, critical thinking, clinical reasoning, clinical decision‐making, and clinical judgement explicit. Second, it offers a framework for linking cognitive processes to competency development and observable professional practice. Third, it provides educators with a shared language and structure for supporting students' development of professional reasoning within the NCP.

These contributions are particularly relevant in undergraduate nutrition and dietetics education, where students are developing foundational understandings of professional reasoning, the NCP, and their emerging professional identity. Although existing frameworks, such as the EFAD Academic Standards and legal regulations for health professions, acknowledge the importance of cognitive capabilities, they provide limited guidance on how these processes can be conceptually integrated, made pedagogically explicit, and translated into teaching and assessment practices [60].

For educators, the model offers a structured basis for curriculum design, case‐based learning, reflective practice, and metacognitive development. Rather than prescribing a specific sequence of action, it encourages students to recognise how different forms of professional thinking interact during nutrition and dietetic care. In this sense, the model serves as a pedagogical tool for making otherwise implicit cognitive processes visible and discussable.

Feedback from nutrition and dietetics educators further highlighted the pedagogical relevance of the model. Participants particularly emphasised its value in clarifying relationships among cognitive strategies and supporting explicit discussion of professional thinking within nutrition and dietetics education. By illustrating how cognitive strategies interact across different levels of practice, the model may help learners understand not only individual clinical decisions but also the broader organisational, social, environmental, and policy contexts that shape nutrition and dietetics care.

The model may also support interprofessional education by providing a common conceptual vocabulary for discussing cognitive processes across disciplinary boundaries. Such a framework may facilitate dialogue about professional thinking, help students recognise commonalities and differences across health professions, and support collaborative approaches to addressing complex nutrition‐related problems.

Although developed within a specific institutional context, the model may hold broader relevance beyond this setting. Its transferability, however, will depend on how the five cognitive strategies are interpreted, aligned with local competency frameworks, and enacted within diverse educational, professional, and regulatory contexts.

### 4.5 International and Cross‐Contextual Adaptation

As the comparison with British, Australian, and US dietetic frameworks illustrates, related cognitive processes may be embedded in different competency domains, practice models, credentialing requirements, and performance expectations. The terminology used for clinical reasoning, decision‐making, judgement, and systems thinking may therefore vary, as may the extent to which these processes are made explicit or assessed independently.

Educators seeking to adapt the *Five Cognitive Strategies in Dietetics Education Pilot Model* should therefore avoid direct terminology‐based transfer. A practical adaptation process could involve mapping the five cognitive strategies against local accreditation, credentialing, and professional competency standards; examining how they are expressed within the locally used dietetic process; and developing context‐specific examples, observable activities, and assessment indicators with educators, practitioners, and students. This would allow the conceptual distinctions within the model to be retained while adapting their educational enactment to local professional, regulatory, and cultural expectations.

### 4.6 Reflections, Limitations, and Future Trajectories

This project contributes to dietetic education by explicitly linking five cognitive strategies that are frequently discussed separately and positioning them within a unified model aligned with the NCP.

However, several limitations and reflexive considerations must be acknowledged. Consistent with its qualitative, iterative–inductive logic [14], the study was exploratory and aimed to generate conceptual clarity rather than generalisable claims. The model was developed through a non‐systematic literature review, consultations with health professions educators, and feedback from nutrition and dietetics educators, with study selection and analytical emphasis guided by the authors' professional judgement. The consultation component involved a small purposive sample of five educators from three health professions within a single institution. As such, observations regarding the relative emphasis placed on specific cognitive strategies reflect the perspectives of individual educators within a particular curricular context and should not be interpreted as representative of the professions more broadly. Consistent with the exploratory aims of the study, the consultations were intended to inform conceptual development. The predominantly dietetic background of the author team shaped methodological choices and interpretive emphasis, as is common in qualitative research [12].

Despite ongoing reflexive practice – understood as ‘collaborative, responsible, iterative, engaged, agile, and creative’ [61] – challenges remained in integrating sometimes contradictory inputs into coherent definitions and a unified model. Regular co‐author discussions supported transparency and consideration of alternative interpretations, while credibility was strengthened through prolonged engagement with multiple data sources and iterative refinement. Nevertheless, these constraints may have affected internal coherence and interpretive depth and highlight areas for methodological strengthening.

Further limitations relate to the model's developmental stage and contextual embeddedness. The alignment of cognitive strategies with the NCP remains conceptual, and neither the model nor its components have been empirically tested or validated, limiting transferability beyond the national Swiss institutional context [13, 62]. Importantly, students – the primary target group – were not involved in the model's development. Future research should therefore include student‐centred enquiry into its effects on learning, metacognition, and professional identity formation [5], as well as collaboration with practice partners to explore how these cognitive strategies can be observed and assessed in practice placements.

A particular point of uncertainty concerns the positioning of systems thinking as the overarching orientation within the model. This positioning was supported primarily by contemporary literature rather than by educator perspectives and therefore warrants further empirical investigation. Future research should examine whether systems thinking is best understood as an overarching orientation, a distinct cognitive strategy, or an integrative capability that operates across multiple dimensions of dietetic practice.

From an educational perspective, further work is needed to develop teachable approaches and assessable indicators for each strategy, with implications for programme‐level assessment and quality assurance. A more systematic and interdisciplinary literature review, including pedagogical research, would also strengthen the evidence base.

A further limitation concerns conceptual selectivity. The five strategies emerged from the local curriculum process, educator consultations, and targeted literature engagement and do not constitute an exhaustive account of cognition or competence in dietetic practice. Other relevant constructs include metacognition, reflective and ethical reasoning, collaborative reasoning, shared decision‐making, strategic competence, and integrated problem‐solving. These constructs may partly overlap with the five strategies but do not necessarily operate at the same conceptual level. Some describe cognitive processes, whereas others refer to professional orientations, relational practices, or observable forms of action. Future development should examine whether these constructs are best represented as additional cognitive strategies, contextual influences, or ways of practising through which the current model is enacted.

Taken together, these limitations reflect both the constraints and opportunities of a pilot model developed alongside ongoing curriculum reform. Continued interdisciplinary collaboration, systematic literature synthesis, and empirical validation involving students, educators, and practitioners will be essential to refine the model into a robust yet adaptable framework capable of responding to evolving professional demands and increasing health system complexity.

Future empirical work should therefore evaluate not only whether students understand the five concepts, but also whether they can integrate them within individual, collaborative, and systems‐level professional activities, and whether EPA‐based approaches can validly support and assess this development. Ultimately, the educational value of the *Five Cognitive Strategies in Dietetics Education Pilot Model* will depend on whether it can help students integrate conceptual understanding, competency development, and professional practice in ways that support effective nutrition and dietetic care within increasingly complex health systems.

## 5 Conclusion

This study explored how key cognitive strategies are conceptualised within and across health professions, and how they can be more coherently articulated in Bachelor‐level nutrition and dietetics education using the NCP as a reference structure. Through an iterative, interpretive process integrating interprofessional consultations, targeted literature engagement, educator feedback, and reflexive discussion, it developed the *Five Cognitive Strategies in Dietetics Education Pilot Model* linking five strategies: systems thinking, critical thinking, clinical reasoning, clinical decision‐making, and clinical judgement.

The model is not intended as a definitive framework but as a context‐specific heuristic educational framework designed to support reflection, teaching, and curriculum development within the NCP. Its principal contribution lies in bridging conceptual understanding, competency development, and professional practice by making relationships among systems thinking, critical thinking, clinical reasoning, clinical decision‐making, and clinical judgement explicit. In doing so, it may foster metacognitive awareness, professional reasoning, and dialogue within both nutrition and dietetics education and interprofessional learning contexts.

As an exploratory, interpretive synthesis, the findings remain preliminary and the model unvalidated. Further work – including pilot implementation, feedback from students and educators, and empirical evaluation – is needed to refine its pedagogical value and assess its applicability beyond the original context.

## Author Contributions

**Sonja Schönberg:** writing – original draft (lead), methodology (equal), analysis (equal), review and editing (equal), project administration. **Elisa Bertozzi:** investigation (equal), analysis (equal), writing – original draft (equal), review and editing (equal). **Jessica Stalder:** investigation (equal), analysis (equal), writing – original draft (equal), review and editing (equal). **Anita Bucher:** analysis (equal), visualisation (lead), review and editing (equal). **Joyce Haddad:** methodology (equal), writing – review and editing (equal). **Adrian Rufener:** conceptualisation (lead), investigation (equal), analysis (equal), writing – review and editing (equal). **Sandra Jent:** conceptualisation (equal), methodology (equal), analysis (equal), writing – original draft, review and editing (equal). This manuscript is an original work and has not been published elsewhere nor is it under consideration by any other journal. All authors have contributed substantially to the conception and development of the study and have approved the final version of the manuscript.

## Funding

The authors have nothing to report.

## Ethics Statement

This research did not involve personal health or other sensitive data and focused solely on collecting expert opinions regarding the suitability of a teaching instrument for quality management and professional development. In accordance with applicable regulations, the study does not fall under the scope of the Swiss Human Research Act (HRA) and therefore did not require formal approval from a cantonal ethics committee. All sources used have been appropriately acknowledged. Informed consent was obtained from all participants and participation was voluntary. The authors declare no conflicts of interest related to this work. We affirm that the manuscript complies with the Journal of Human Nutrition and Dietetics' Guidelines on Publishing and Research Ethics.

## Conflicts of Interest

The authors declare no conflicts of interest.

## 2 Transparency Declaration

The lead author confirms that this manuscript provides an honest, accurate, and transparent account of the study reported. No essential aspects of the study have been omitted, and any deviations from the original plan have been fully explained.

## Supporting information


Supporting File

## Data Availability

The data that support the findings of this study are available from the corresponding author upon reasonable request.
