# Supplementary material for: Integrating Systems and Critical Thinking, Clinical Reasoning, Decision‐Making, and Judgement in Dietetic Education: Development of a Conceptual Pilot Model
Source: J Hum Nutr Diet. 2026 Sep 22;39(5):e70343. doi: 10.1111/jhn.70343 (PMC13598357; doi:10.1111/jhn.70343)
Supplement: Supplementary file 1 [file JHN-39-0-s001.docx]

Table S1: Key literature informing the conceptualisation of the five cognitive strategies included in the study.

| Reference | Cognitive Strategy | | | | | Conceptual and educational contribution | | | |
| --- | --- | --- | --- | --- | --- | --- | --- | --- | --- |
|  | Critical Thinking | Clinical Reasoning | Clinical Decision Making | Clinical Judgement | Systems Thinking | Conceptualisation | Contextualisation | Implementation in nutrition and dietetics | Education |
| Amissah et al., 2020 [1] |  |  |  |  | x | x |  |  | x |
| Bergquist et al., 2024  [2] |  |  |  |  | x |  | x | x | x |
| Bergquist et al., 2025  [3] |  |  |  |  | x | x | x | x | x |
| Bergquist & Tagtow, 2026  [4] | x |  |  |  | x | x | x | x | x |
| Charney & Peterson, 2013  [5] | x |  |  |  |  | x | x | x | x |
| Chin‐Yee & Upshur, 2018  [6] |  |  |  | x |  | x | x |  | x |
| Commission on Dietetic Registration., n.d.  [7] | x |  |  |  |  | x |  |  | x |
| Connor et al., 2023  [8] |  |  |  | x |  | x | x |  |  |
| Diamond-Fox & Bone, 2021  [9] | x | x |  |  |  | x | x |  |  |
| Dolansky et al., 2020  [10] |  |  |  |  | x | x |  |  |  |
| Elangovan et al., 2018  [11] | x |  | x |  |  | x | x |  |  |
| Facione, 2015  [12] | x |  |  |  |  | x | x |  | x |
| Gadsby & Wilding, 2024  [13] |  |  |  |  | x | x |  |  |  |
| Gonzalez & Nielsen, 2024  [14] |  | x |  | x |  |  | x |  | x |
| Holder, 2018  [15] |  | x |  |  |  | x | x |  | x |
| Indrašienė et al., 2023  [16] | x |  |  |  |  |  |  |  | x |
| Kienle & Kiene, 2011 [17] |  |  |  | x |  | x | x |  |  |
| Klemme & Siegmann, 2015  [18] | x | x | x |  |  | x | x |  |  |
| Lateef, 2018  [19] | x | x | x |  |  | x | x |  | x |
| Linn et al., 2012  [20] |  | x |  |  |  | x | x |  | x |
| Marino et al., 2020  [21] |  |  | x |  |  | x | x |  |  |
| Monat & Gannon, 2015  [22] |  |  |  |  | x | x |  |  |  |
| Patton, 2010  [23] |  |  | x |  |  | x |  |  |  |
| Rufener & Jent, 2016  [24] | x |  |  |  |  | x |  | x | x |
| Stalter et al., 2017  [25] |  |  |  |  | x | x | x |  | x |
| Swan et al., 2017  [26] | x |  |  |  |  | x | x | x | x |
| Tagtow et al., 2022  [27] |  |  |  |  | x | x | x | x | x |
| Tagtow et al., 2024  [28] |  |  |  |  | x | x | x | x |  |
| Tagtow et al., 2025  [29] |  |  |  |  | x | x | x | x |  |
| Trbovich, 2014  [30] |  |  |  |  | x | x | x |  |  |
| Vo et al., 2020  [31] |  |  | x | x |  | x | x |  |  |
| Vo et al., 2021  [32] |  |  |  | x |  | x | x | x | x |

# References

1.	Amissah M, Gannon T, Monat J. What is Systems Thinking? Expert Perspectives from the WPI Systems Thinking Colloquium of 2 October 2019. Systems. 2020;86:6. doi:10.3390/systems8010006

2.	Bergquist EE, Buckingham-Schutt L, Smalley S, Campbell C, Dollisso A, Qu S. Nutrition and Dietetics Educators’ Experiences Using Systems Thinking in Teaching. J Diet Educ. 2024;22:111–26. doi:10.26890/vykl2341

3.	Bergquist EE, Buckingham‐Schutt L, Smalley S, Campbell CG, Dollisso A, Qu S. Integrating Systems Thinking in Nutrition and Dietetics Education. J Hum Nutr Diet. 2025;382. doi:10.1111/jhn.70036

4.	Bergquist EE, Tagtow AM. Strategies for Systems Thinking and Sustainable Food Systems Integration in Dietetics Education. J Acad Nutr Diet. 2026;1261:156195. doi:10.1016/j.jand.2025.08.006

5.	Charney P, Peterson SJ. Practice Paper of the Academy of Nutrition and Dietetics: Critical Thinking Skills in Nutrition Assessment and Diagnosis. J Acad Nutr Diet. 2013;11311. doi:10.1016/j.jand.2013.09.006

6.	Chin‐Yee B, Upshur R. Clinical judgement in the era of big data and predictive analytics. J Eval Clin Pract. 2018;243:638–45. doi:10.1111/jep.12852

7.	Commission on Dietetic Registration. Critical Thinking Self-Study Evaluation Tool: An Introduction [Internet]. [cited 2026 Mar 8]. Available from: https://admin.cdrnet.org/vault/2459/web/Critical%20Thinking%20Evaluation%20Form%20Final-NEW1.pdf

8.	Connor J, Flenady T, Massey D, Dwyer T. Clinical judgement in nursing – An evolutionary concept analysis. J Clin Nurs. 2023;3213–14:3328–40. doi:10.1111/jocn.16469

9.	Diamond-Fox S, Bone H. Advanced practice: critical thinking and clinical reasoning. Br J Nurs. 2021;309:526–32. doi:10.12968/bjon.2021.30.9.526

10.	Dolansky MA, Moore SM, Palmieri PA, Singh MK. Development and Validation of the Systems Thinking Scale. J Gen Intern Med. 2020;358:2314–20. doi:10.1007/s11606-020-05830-1

11.	Elangovan S, Guzman-Armstrong S, Marshall TA, Johnsen DC. Clinical decision making in the era of evidence-based dentistry. J Am Dent Assoc. 2018;1499:745–7. doi:10.1016/j.adaj.2018.06.001

12.	Facione PA. Critical Thinking: What It Is and Why It Counts [Internet]. Insight Assessment; 2015 [cited 2026 Mar 8]. Available from: https://insightassessment.com/iaresource/critical-thinking-what-it-is-and-why-it-counts/

13.	Gadsby EW, Wilding H. Systems thinking in, and for, public health: a call for a broader path. Health Promot Int. 2024;394. doi:10.1093/heapro/daae086

14.	Gonzalez L, Nielsen A. An integrative review of teaching strategies to support clinical judgment development in clinical education for nurses. Nurse Educ Today. 2024;133:106047. doi:10.1016/j.nedt.2023.106047

15.	Holder AG. Clinical Reasoning: A State of the Science Report. Int J Nurs Educ Scholarsh. 2018;151. doi:10.1515/ijnes-2016-0024

16.	Indrašienė V, Jegelevičienė V, Merfeldaitė O, Penkauskienė D, Pivorienė J, Railienė A, et al. Critical Reflection in Students’ Critical Thinking Teaching and Learning Experiences. Sustainability. 2023;1518:13500. doi:10.3390/su151813500

17.	Kienle GS, Kiene H. Clinical judgement and the medical profession. J Eval Clin Pract. 2011;174:621–7. doi:10.1111/j.1365-2753.2010.01560.x

18.	Klemme B, Siegmann G, editors. Clinical Reasoning: Therapeutische Denkprozesse lernen. 2nd ed. Stuttgart: Georg Thieme Verlag; 2015. doi:10.1055/b-002-101352

19.	Lateef F. Clinical Reasoning: The Core of Medical Education and Practice. Int J Intern Emerg Med. 2018;12:1015.

20.	Linn A, Khaw C, Kildea H, Tonkin A. Clinical reasoning: A guide to improving teaching and practice. Aust Fam Physician. 2012;411:18–20.

21.	Marino MA, Andrews K, Ward J. Clinical Decision Making at the Bedside. Nurs Clin North Am. 2020;551:29–37. doi:10.1016/j.cnur.2019.10.003

22.	Monat JP, Gannon TF. What is Systems Thinking? A Review of Selected Literature Plus Recommendations. Am J Syst Sci. 2015;41:11–26. doi:10.5923/j.ajss.20150401.02

23.	Patton DD. Introduction to Clinical Decision Making. Semin Nucl Med. 2010;405:319–26. doi:10.1053/j.semnuclmed.2010.05.001

24.	Rufener A, Jent S, editors. Der Ernährungstherapeutische Prozess: Lehrbuch für Studium und Praxis. 1. Auflage. Göttingen: Hogrefe; 2016.

25.	Stalter AM, Phillips JM, Ruggiero JS, Scardaville DL, Merriam D, Dolansky MA, et al. A Concept Analysis of Systems Thinking. Nurs Forum Auckl. 2017;524:323–30. doi:10.1111/nuf.12196

26.	Swan WI, Vivanti A, Hakel-Smith NA, Hotson B, Orrevall Y, Trostler N, et al. Nutrition Care Process and Model Update: Toward Realizing People-Centered Care and Outcomes Management. J Acad Nutr Diet. 2017;11712:2003–14. doi:10.1016/j.jand.2017.07.015

27.	Tagtow AM, Herman D, Cunningham-Sabo L. Next-Generation Solutions to Address Adaptive Challenges in Dietetics Practice: The I+PSE Conceptual Framework for Action. J Acad Nutr Diet. 2022;1221:15–24. doi:10.1016/j.jand.2021.01.018

28.	Tagtow AM, Welter C, Seweryn S, Spiker ML, Lange J, Asada Y. The intersection of systems thinking and structural empowerment in the work of public health dietitians. J Hum Nutr Diet. 2024;376:1475–85. doi:10.1111/jhn.13372

29.	Tagtow AM, Welter CR, Seweryn S, Spiker ML, Lange J, McDonald J, et al. Enhancing Adaptability: Exploring Structural Empowerment and Systems Thinking Among State Governmental Public Health Nutritionists. J Public Health Manag Pract. 2025;311:E20–5. doi:10.1097/PHH.0000000000002026

30.	Trbovich P. Five Ways to Incorporate Systems Thinking into Healthcare Organizations. Biomed Instrum Technol. 2014;48s2:31–6. doi:10.2345/0899-8205-48.s2.31

31.	Vo R, Smith M, Patton N. A model of the multidimensional nature of experienced dietitian clinical decision-making in the acute care setting. J Hum Nutr Diet. 2020;335:614–23. doi:10.1111/jhn.12756

32.	Vo R, Smith M, Patton N. The role of dietitian clinical judgement in the nutrition care process within the acute care setting: a qualitative study. J Hum Nutr Diet. 2021;34:124–33. doi:10.1111/jhn.12820
